# Synergistic Density Functional Theory and Molecular Dynamics Approach to Elucidate PNIPAM–Water Interaction Mechanisms

**DOI:** 10.3390/ma18112498

**Published:** 2025-05-26

**Authors:** Noor Alomari, Santiago Aparicio, Paul Meyer, Yi Zeng, Shuang Cui, Alberto Gutiérrez, Mert Atilhan

**Affiliations:** 1 Chemical and Paper Engineering Department, Western Michigan University, Kalamazoo, MI 49008, USA; noor.f.alomari@wmich.edu; 2 Department of Chemistry, University of Burgos, 09001 Burgos, Spain; sapar@ubu.es; 3 National Renewable Energy Laboratory, 15013 Denver West Parkway, Golden, CO 80401, USA; paul.meyer@nrel.gov (P.M.); yi.zeng@nrel.gov (Y.Z.); 4 Department of Mechanical Engineering, University of Texas at Dallas, 800 W Campbell Rd, Richardson, TX 75080, USA

**Keywords:** poly(N-isopropylacrylamide) (PNIPAM), water sorption, hydrogen bonding interactions, DFT calculations, MD (LAMMPS) simulations, lower critical solution temperature

## Abstract

This study employs Density Functional Theory (DFT) and Molecular Dynamics (MD) simulations to investigate interactions between water molecules and Poly(N-isopropylacrylamide) (PNIPAM). DFT reveals preferential water binding sites, with enhanced binding energy observed in the linker zone. Quantum Theory of Atoms in Molecules (QTAIM) and electron localization function (ELF) analyses highlight the roles of hydrogen bonding and steric hindrance. MD simulations unveil temperature-dependent hydration dynamics, with structural transitions marked by changes in the radius of gyration (Rg) and the radial distribution function (RDF), aligning with DFT findings. Our work goes beyond prior studies by combining a DFT, QTAIM and MD simulations approach across different PNIPAM monomer-to-30mer structures. It introduces a systematic quantification of pseudo-saturation thresholds and explores water clustering dynamics with structural specificity, which have not been previously reported in the literature. These novel insights establish a more complete molecular-level picture of PNIPAM hydration behavior and temperature responsiveness, emphasizing the importance of amide hydrogen and carbonyl oxygen sites in hydrogen bonding, which weakens above the lower critical solution temperature (LCST), resulting in increased hydrophobicity and paving the way for understanding water sorption mechanisms, offering guidance for future applications such as dehumidification and atmospheric water harvesting.

## 1. Introduction

Moisture control and efficient water removal are critical in chemical processes. The presence of unwanted moisture can significantly alter reaction kinetics, compromise the purity of products and degrade sensitive materials. Moreover, poor humidity control and water drying can increase energy consumption, resulting in a higher carbon footprint. Thus, developing materials that effectively manage humidity and expedite water removal is essential for the reliability, safety and environmental sustainability of chemical processes. This need places such materials at the forefront of innovations in the chemical industry.

Porous materials like silica gel, zeolites, activated carbon and metal–organic frameworks (MOFs) have been widely studied as potential ad/absorbents [1] due to their high surface area-to-volume ratios as well as their capacity to absorb, retain and transport large volumes of water molecules within a short timeframe [2]. However, limitations such as hydrolytic and structural instability hinder their scalability and long-term use [3,4]. In the search for better alternatives, certain hydrophilic polymers have shown promise due to their superior water ad/absorption capacities, outperforming traditional porous materials such as silica gel and zeolites. These polymers interact with water via specific sites between macromolecular chains, resulting in the swelling of the polymeric network. Nevertheless, their regeneration still relies on energy-intensive evaporation.

More recently, attention has been focused on thermoresponsive polymers, a category of polymers characterized by lower critical solution temperatures (LCSTs). This LCST property offers potential enhanced drying applications due to their ability to respond to small changes in temperature. These materials exhibit hydrophilic behavior below the LCST and become hydrophobic above it, enabling controlled water release. Among them, poly(N-isopropylacrylamide) (PNIPAM) is a classic, well-studied example [5,6]. The adsorption and desorption processes of PNIPAM can be easily altered through slight temperature changes due to its inherent thermal sensitivity [7]. PNIPAM undergoes a significant volume phase transition at roughly 32 °C (its LCST), with a corresponding shift in the solubility and swelling ratio, leading to potential applications in the creation of stimulus-responsive emulsions [8]. This transition is relevant in developing smart systems for responsive water control. While PNIPAM gels exhibit complex swelling behavior, linear chains also reveal critical hydration dynamics on nanoscopic scales. Understanding these conformational changes and hydration interactions across the LCST is key to optimizing its use in drying and moisture-responsive systems.

Transitioning to a nanoscopic viewpoint presents even more challenges but also reveals intricate details of the system’s behavior. Recent studies highlight the role of non-equilibrium effects due to surface heterogeneities and interface dynamics [9,10]. These findings motivate a more detailed investigation into the thermodynamics and interaction mechanisms between PNIPAM and water at the molecular level.

In this work, we investigate the structural behavior of PNIPAM and its copolymers and their dynamic interactions with water molecules. Understanding these interactions is key to optimizing PNIPAM for efficient water harvesting. By integrating Density Functional Theory (DFT), Quantum Theory of Atoms in Molecules (QTAIM) and Molecular Dynamics (MD) simulations, we have explored the water–polymer interaction landscape across various isothermal conditions [11,12]. This synergistic approach reveals water binding sites, saturation limits, and the tunable hydrophilic–hydrophobic balance of PNIPAM. By analyzing the chemical structures from the monomer to 30mer scale, we identify steric and electronic factors affecting water ab/adsorption and uncover temperature-dependent hydration dynamics. By uncovering the complex forces governing PNIPAM–water interactions, this work offers a framework for designing advanced polymers to accelerate material development and enable transformative applications in water treatment, agriculture, biomedical engineering, and related fields. In contrast to previous studies that have primarily examined bulk swelling or isolated functional group interactions, our integrated DFT, QTAIM and MD approach systematically examines hydration behavior across PNIPAM monomer-to-30mer structures. We uncover saturation thresholds, steric limitations and temperature-dependent hydration dynamics with unprecedented spatial and energetic resolution, offering insights into hydration mechanisms not previously resolved in the literature.

## 2. Computational Methods

### 2.1. Density Functional Theory (DFT)

PNIPAM and its water mixture systems were studied using first principles by performing DFT calculations using ORCA software (Version 4.2.1) [13]. The systems considered were (*i*) a PNIPAM monomer repeat unit; (*ii*) a PNIPAM monomer repeat unit with only one water molecule, considering different possible interaction sites; (*iii*) a PNIPAM monomer repeat with several water molecules localized at the highest binding energy site; (*iv*) a PNIPAM oligomer (3mer) with various water molecules at potential active sites; and (*v*) the sites that showed the most-active water affinity within the PNIPAM backbone and the 5, 10, 15 and 20 water molecules located around this segment. Smaller and discrete clusters (5, 10, 15 and 20 water molecules located around PNIPAM monomer and 3mer structures) were used to incrementally probe hydration effects at the monomer and oligomer (3mer) levels. These numbers of water molecules were selected to capture the stepwise evolution of the hydration shell and interaction energy trends while maintaining computational tractability. The most stable structure for a PNIPAM monomer was obtained alone before combining to form the NIPAM 3mer and exposing it to water molecules in each of the described cases above. The initial geometry of the PNIPAM monomer, PNIPAM 3mer, and water molecule was constructed using Avogadro molecular modeling software [14], version 2.0 (GPLv2). Geometry optimizations for PNIPAM and water were carried out using a B3LYP functional in conjunction with the 6-311++G** basis set [15]. In contrast, for the more complex systems involving the PNIPAM trimer and its hydrated form, geometry optimizations were performed using the BLYP functional in combination with the 6-311++G** basis set [16]. In our study, B3LYP was initially employed for the monomer-level calculations due to its well-established accuracy in predicting molecular geometries and non-covalent interaction energies. However, as the system size increased (particularly for the 3mer and 3mer with water cases), the computational cost associated with hybrid functionals such as B3LYP and M06-2X became prohibitive. Therefore, BLYP was chosen for these larger systems due to its favorable trade-off between computational efficiency and accuracy in modeling hydrogen-bonded and dispersion-dominated interactions, especially when used in conjunction with the 6-311++G** basis set and counterpoise correction. The literature has reported that BLYP performs reliably for non-covalent interactions in structurally analogous systems, such as peptides [17] or boron-based aromatic systems and β-cyclodextrin [18]. Furthermore, although the absolute energy values obtained from B3LYP and BLYP may differ, our analysis focuses on relative trends in interaction strength, electron density topologies and hydrogen bonding features across different structural regions and hydration levels. These trends are generally conserved across functionals within the DFT framework, ensuring the comparability of key insights. Notably, both BLYP and B3LYP provide qualitatively similar descriptions of hydrogen bonding and electrostatic interactions in organic systems, as discussed in the benchmark studies by Zhao and Truhlar [19]. Thus, the comparability of our findings is maintained at the trend and interpretation level, which is the focus of this work. The isolated monomer and water molecules were initially optimized separately, after which they were combined in different spatial arrangements. For each configuration, the structure with the lowest final optimized energy from DFT calculations was selected as the most stable. Following optimization, the interaction energies for the final monomer–water complexes (*E_M-W_*) were calculated using the following expression:

*E*_*M*-*W*_ = *E*_*M*-*W*_ − (*E*_*M*_ + *E*_*W*_) (1)

All energy calculations accounted for basis set superposition error (BSSE) using the counterpoise correction method [20]. Typically, interaction energies between two species, A and B, are determined as the difference between the total energy of the complex (AB) and the sum of the energies of the isolated components. However, the choice of basis set can limit the accurate representation of dispersion interactions. To mitigate this limitation and improve the reliability of the interaction energy estimates, counterpoise correction was applied. The BSSE-corrected interaction energy is calculated as follows:

*E*_*int*_ = (*E*_*AB*_)^*AB*^ − [(*E*_*A*_)^*A*^ + (*E*_*B*_)^*B*^] (2)

The superscripts AB denote that the energies of both the complex and the individual components are computed using the same complete basis set. To calculate the energy of component A in the presence of the mixture basis, the basis functions of component B are included at its atomic positions, but without incorporating its electrons or nuclear charges. This approach, known as the use of “ghost atoms”, is a key feature of the counterpoise correction method. These calculations were performed during the post-DFT analysis, and the resulting values are reported in this study. It should be noted that neither an error estimation nor a sensitivity analysis for binding energies at this level of theory have been included, as they reflect deterministic outcomes from a given functional and basis set. However, numerical stability was ensured by using tight self-consistent field (SCF) convergence criteria, employing counterpoise correction (to account for basis set superposition error, BSSE, in all interaction energy calculations) and validating trends across multiple hydration states. The final optimized geometries of all investigated systems were obtained, and the lowest energy configurations of PNIPAM and its complexes with water were selected for detailed analysis of intermolecular interactions. These interactions were characterized using QTAIM descriptors, including (*i*) the identification of bond critical points (BCPs) and ring critical points (RCPs), along with their corresponding electron density (*ρ*) and the Laplacian of the electron density (∇^2^*ρ*); (*ii*) the visualization of the reduced density gradient (RDG) isosurfaces; (*iii*) electrostatic potential (ESPs) maps; and (*iv*) electron localization functions (ELFs). Furthermore, electronic properties such as density of states (DOS) and HOMO-LUMO energy gaps were also examined. DOS plots were generated from the distribution of molecular orbital energy levels, where the highest occupied molecular orbital (HOMO) represents the topmost filled level, and the lowest unoccupied molecular orbital (LUMO) denotes the bottommost empty level. The polymer backbone segment exhibiting the strongest interaction with water was further analyzed to assess the nature of intermolecular interactions and provide insight into the saturation behavior of water in the PNIPAM system. These topological characterizations were performed according to the QTAIMs (Bader’s AIM theory) [21] by using the MultiWFN program (Version 3.8) [22]. In all cases, atomic charges were determined from the optimized geometries using the ChelpG scheme [23], as implemented in the ORCA computational package, version 6.0.0.

### 2.2. Molecular Dynamics (MD)

Molecular Dynamics (MD) simulations were conducted using the open-source software LAMMPS (Large-scale Atomic/Molecular Massively Parallel Simulator) [11], stable release 2 version, August 2023. The simulations were performed within a cubic simulation box measuring 70 × 30 × 30 Å^3^, with periodic boundary conditions (PBCs) applied along all three spatial dimensions. These dimensions were set to maintain a water density close to 1 g/cm^3^ after solvation, ensuring no artificial crowding of the PNIPAM 30mer structure or excessive vacuum regions. This approach is standard in solvated polymer simulations, and we confirmed that no significant box-size effects distorted PNIPAM 30mer structure–water interactions or the hydration structure during the trajectory. The starting polymeric configuration was prepared using Moltemplate software (version 2023.2.6) [24]. Bond stretching, van der Waals forces, dihedral torsions and angle bending interactions were all accounted for using parameters from the united-atom OPLS-AA force field [25]. The thirty repeat unit monomer segment (30mer) structure was placed in the box with 5000 SPC/E model water molecules [26,27]. We used 5000 water molecules to reproduce an experimentally relevant water density (~1 g/cm^3^) within a periodic simulation box large enough to minimize boundary effects and ensure the solvation of the PNIPAM 30mer structure under ambient conditions. The initial configurations were subjected to energy minimization using the conjugate gradient method for 2000 steps. The polymer backbone was maintained in a fully extended and rigid conformation throughout the energy minimization process. Following minimization, water equilibration was carried out in the NPT (isothermal-isobaric) ensemble (at temperatures ranging from 293 to 323 K and a pressure of 1 bar). This equilibration phase employed a Nose–Hoover thermostat and barostat to regulate temperature and pressure [28], ensuring the proper optimization of the polymeric system.

Non-bonded interactions, including van der Waals forces, were represented using Lennard-Jones potential. Likewise, to account for all possible interaction types, a geometric mixing rule (“pair modify”) was applied to estimate missing Lennard-Jones parameters. A cutoff distance of 12.0 Å was used for both the real-space component of the electrostatic interactions and the van der Waals forces. Coefficients for the “special bonds” command in LAMMPS were set as a combination of Lennard-Jones and columbic forces at a value of 0.5 for all simulations [11].

The total energy of the force field consisted of four main contributions: bond stretching, angle bending, dihedral angle torsion, and non-bonded interactions. The first three components are collectively referred to as bonded interactions, and their functional forms are given by:

*E* = *E*_*bond*_ (*r*) + *E_angle_* (*θ*) + *E_dihedral_* (*ϕ*) + *E_non-bonding_* (3)

*E*_*bond*_ (*r*) = *K_b_* (*r* − *r*_0_)^2^ (4)

*E*_*angle*_ (*θ*) = *K_θ_* (*θ* − *θ*_0_)^2^ (5)

*E_dihedral_* (*ϕ*) = ½ *K*_1_ [1 + *cos*(*φ*)] + ½ *K*_2_ [1 − *cos*(2*φ*)] + ½ *K*_3_ [1 + *cos*(3*φ*)] + ½ *K*_4_ [1 − *cos*(4*φ*)] (6)

Here, *K_b_*, *K_θ_*, *K*_1_, *K*_2_, *K*_3_, and *K*_4_ represent the stiffness constants, while *r*_0_ and *θ*_0_ denote the equilibrium bond length and bond angle, respectively. The expression for non-bonded interactions is defined as follows:

(7) $$E_{non\text{-}bonding}=\sum_{i=1}\sum_{j=i+1}{\left(4\varepsilon_{ij\;}[(\sigma_{ij}/r_{ij}\right)}^{12}-{\left(\sigma_{ij}/r_{ij}\right)}^{6}]+(q_{i}q_{j})/(4\pi \varepsilon_{0}r_{ij}))$$

where *r_ij_* denotes the distance between a pair of atoms, which must be less than the specified cutoff distance *R*. The parameter *ε* corresponds to the depth of the potential energy well, while *σ* indicates the interatomic distance at which the potential energy is zero. The parameters mentioned above were previously established within the OPLS-AA all-atom force field [29]. Finally, the long-ranged electrostatic and van der Waals interactions among charged species were treated using the particle–particle–particle–mesh (PPPM) algorithm (conjugate gradient, CG, method) with an RMS accuracy of 10^−5^ [30]. The bond lengths and the angles of water molecules were constrained using the SHAKE algorithm [31]. The total duration of the simulation was defined as 20 ns (minimization + equilibration + production steps in the NPT ensemble). This time was chosen because in the MD simulations it was observed that the PNIPAM 30mer structure reached equilibrium hydration states and conformational stability within 10–15 ns in the temperature range studied. Full equilibration was confirmed by monitoring total energy, temperature and the radius of gyration vs. simulation time, all of which remained constant. Additional time would not have meaningfully altered the structural or dynamical properties discussed. Finally, it is worth mentioning that the radius of gyration (Rg) determined from the MD simulations and related observables were computed from a single production trajectory, and the standard deviation bars shown in the results represent temporal fluctuations over the last 5–10 ns of the 20 ns simulations, after equilibration. Due to the high computational cost of simulating solvated PNIPAM chains across a range of oligomer lengths and hydration levels, it was decided not to perform multiple independent replicates. While this limits formal statistical inference, the time-averaged properties over equilibrated trajectories offer reliable trends and conformational insights that are consistent with prior studies of polymer-solvent systems.

## 3. Results and Discussion

### 3.1. Initial Geometry of PNIPAM Monomer and Theory Selection Optimization

**Figure 1:**
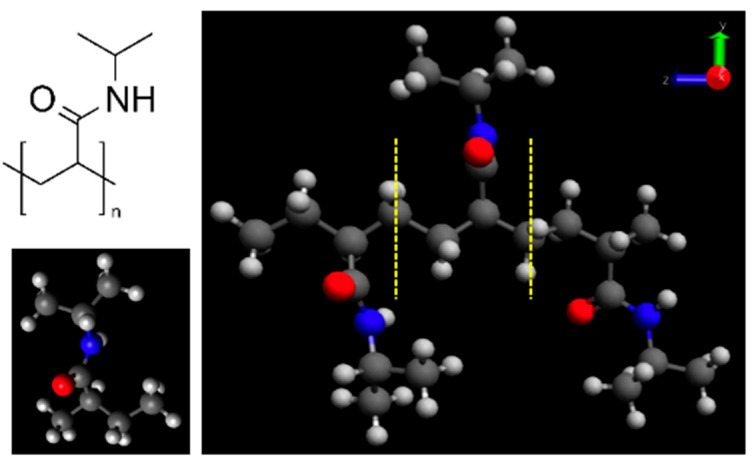
(**left**) PNIPAM repeat unit structure; (**right**) 3mer version of PNIPAM.

**Figure 2:**
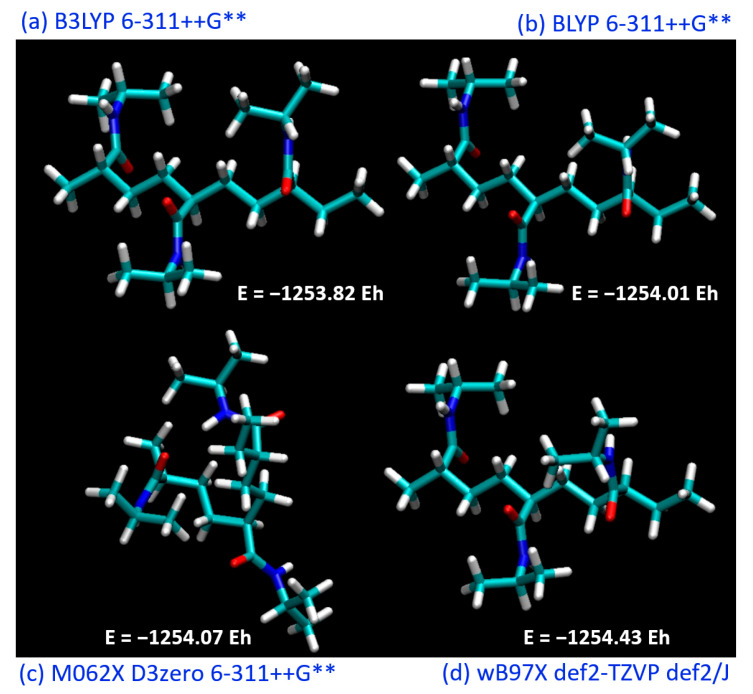
Optimized structure of PNIPAM (3mer) at different theory levels: (**a**) B3LYP, (**b**) BLYP, (**c**) M062X, and (**d**) wB97X.

The PNIPAM monomer and its 3mer version structures are provided in Figure 1. Figure 2 illustrates our preliminary exploration into the structural optimization of the PNIPAM repeat unit under various computational models and force fields.

Our initial investigations constituted the pursuit of an optimal theoretical framework to successfully simulate and evaluate the structure and energetics of a PNIPAM repeat unit. Recognizing the essential role that the appropriate selection of the functional and basis set plays in DFT calculations, we sought to identify the most suitable combination to further our study.

The simulations included a range of commonly employed functionals, including B3LYP, BLYP, M062X, and wB97X. These functionals represent various approaches to the exchange-correlation problem in DFT and offer different degrees of accuracy in predicting molecular properties. Each functional was utilized in conjunction with a variety of basis sets to evaluate their performance in the energy minimization process and to assess their aptitude in capturing the underlying molecular interactions within PNIPAM.

**Figure 3:**
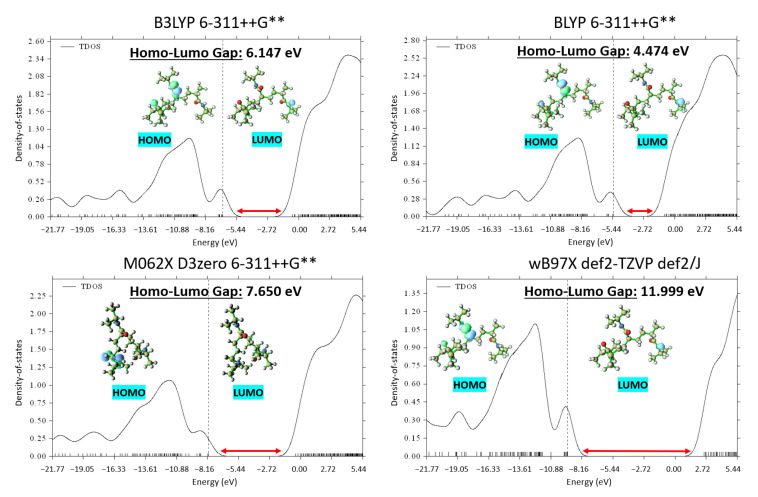
DOS comparative analysis across various theory levels displaying optimal highest-occupied-molecular-orbital–lowest-unoccupied-molecular-orbital gap (HOMO—highest occupied molecular orbital, LUMO—lowest unoccupied molecular orbital).

Although the BLYP functional did not yield the absolute lowest energy compared to the other investigated functionals, it demonstrated an intriguing behavior when considered in conjunction with the DOS analysis. Figure 3 exhibits the DOS plots generated for the PNIPAM repeat unit optimized using the different functionals. The DOS plots provide a clear depiction of the electronic energy level distribution within the system. A particular focus was drawn to the highest-occupied-molecular-orbital–lowest-unoccupied-molecular-orbital gap, a key indicator of the system’s stability and a primary metric for the electronic properties of the material.

Upon evaluation of the highest-occupied-molecular-orbital–lowest-unoccupied-molecular-orbital gaps, the most stable structure was found to emerge from the use of BLYP, evidenced by the smallest energy gap of 4.474 eV. Although this result was not anticipated based solely on total energy calculations, it underscores the importance of employing multiple evaluation criteria when studying complex systems such as PNIPAM. It elucidates how an appropriate functional and basis set cannot solely be determined through energy minimization but must also consider the electronic structure of the material.

Given the superiority of the BLYP functional in terms of the DOS analysis, we elected to adopt this functional for our subsequent DFT simulations. Despite not yielding the minimum energy in the initial optimization, the ability of BLYP to yield the most stable electronic structure in terms of the highest-occupied-molecular-orbital–lowest-unoccupied-molecular-orbital (HOMO-LUMO) gap provides a more robust and reliable foundation for our following in-depth investigations into the intricate behavior of PNIPAM. The identification of the appropriate computational framework in these early stages ensures a solid theoretical grounding for our ongoing exploration of PNIPAM’s water sorption mechanism, structural transformations, and implications for water-harvesting applications.

### 3.2. Identification of Optimal Water Interaction Sites on PNIPAM Oligomer Structure

**Figure 4:**
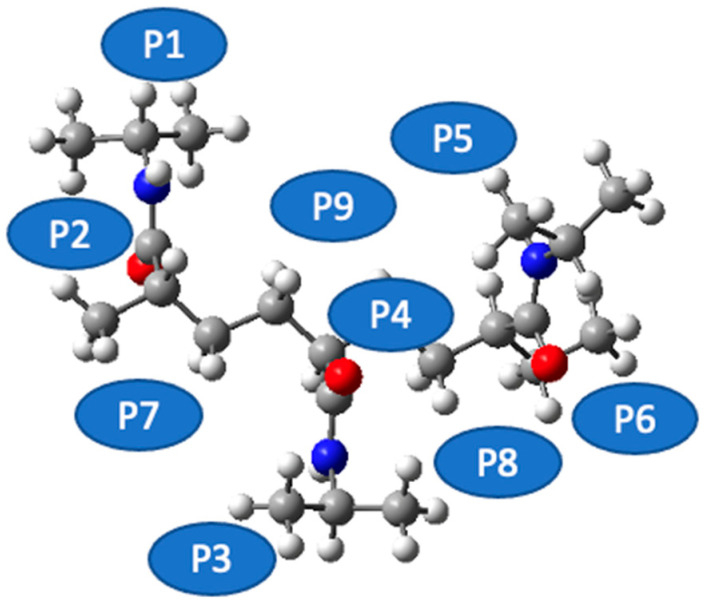
Identification of potential water interaction sites on PNIPAM 3mer structure.

Figure 4 sets out a systematic depiction of the PNIPAM 3mer structure, wherein we have identified and illustrated nine distinct spatial positions for the potential placement of a single water molecule. This meticulous spatial mapping of potential interaction sites establishes a prerequisite for our subsequent analysis of binding energetics and interaction dynamics between water and the PNIPAM oligomer.

As we progress in our computational exploration of water sorption potential, it becomes indispensable to discern the loci on the PNIPAM oligomer that exhibit the most promising affinity for water molecules. Such an understanding would not only reveal key insights into the water–PNIPAM interaction dynamics but also provide a focused framework to delve deeper into ESP distributions, topological analysis, and other post-DFT evaluations, thereby paving the way for a more nuanced understanding of the water sorption mechanism in PNIPAM.

**Figure 5:**
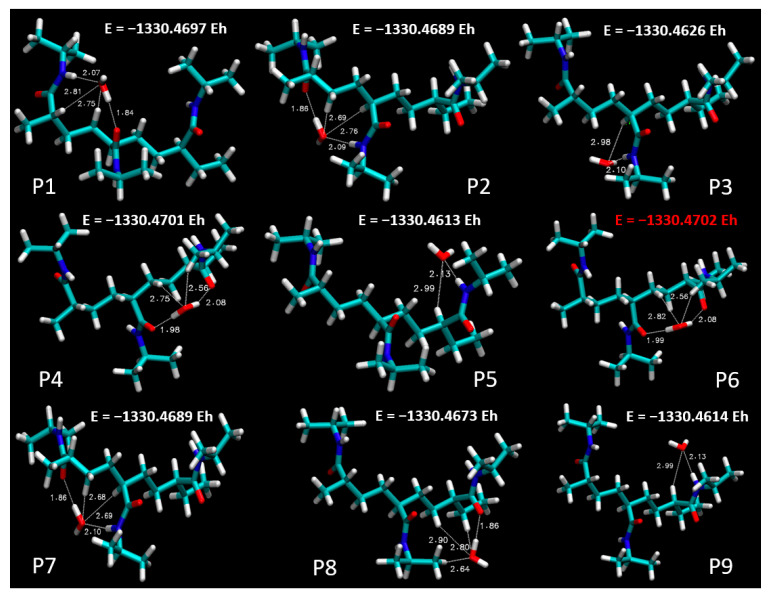
Total energy profiles at distinct spatial positions on PNIPAM 3mer structure.

In pursuit of this goal, we undertook a comprehensive evaluation of the binding energies at each of the demarcated positions on the PNIPAM 3mer structure. The resulting binding energy profiles for the nine positions are presented in Figure 5. These findings offered an unequivocal insight into the preferential sites for water interaction on the PNIPAM 3mer structure. Among the nine distinct spatial positions investigated, Position 6 emerged with the most favorable binding energy for water, registering a value of total energy of −1330.4702 E_h_. This significant binding energy reveals the high affinity of the site at Position 6 for water, suggesting a promising potential for water sorption and retention.

The identification of Position 6 as the site of the highest binding energy underscores its critical role in water–PNIPAM interactions. This insight streamlines the forthcoming investigation into a more focused examination of the structural and electronic features surrounding Position 6. Such a targeted approach enhances the efficiency of our analysis and provides a stronger platform to draw definitive conclusions about the mechanisms underlying the unique water sorption behavior of thermosensitive polymers like PNIPAM. The findings from this segment of our investigation lay a strong foundation for the subsequent analyses. Moving forward, we will focus on investigating the ESP distribution, performing topological characterizations, and conducting other pertinent post-DFT evaluations, specifically around the highest binding energy site, Position 6, on the PNIPAM 3mer structure. Such comprehensive analyses are primed to further elucidate the multifaceted interactions between water and PNIPAM and bring us a step closer to optimizing these polymers for efficient water harvesting.

### 3.3. Quantum Mechanical Characterization of Hydrogen Bonding in Trimer PNIPAM–Water Complex

In an intricate narrative of quantum mechanics and molecular interactions, we delve further into our study of the binding site dynamics for a single water molecule on the trimer PNIPAM structure, employing the remarkable capabilities of the QTAIM. This quantum-level analysis enhances our understanding of the bonding mechanisms in action between the water molecule and the PNIPAM structure and allows us to fathom a more detailed view of this interaction. 

**Figure 6:**
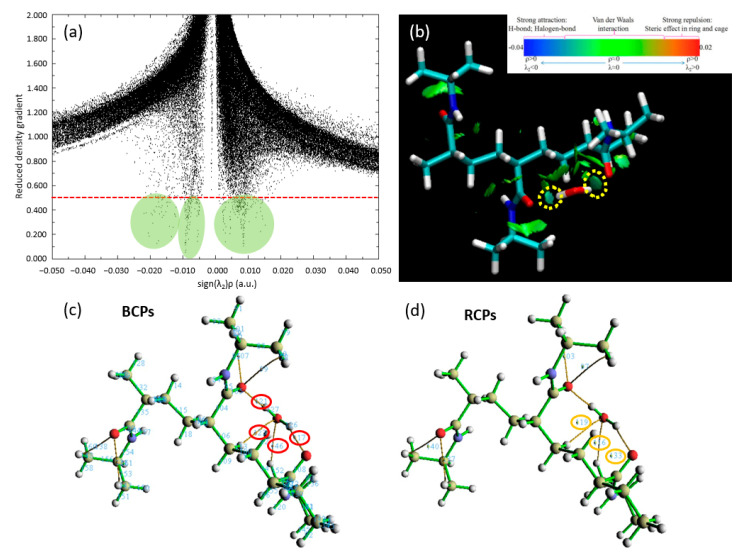
Visualization and quantification of interatomic interactions in the trimer PNIPAM–water complex: insights from QTAIM analysis. (**a**) RDG scatter plot; (**b**) Non-Covalent Interactions (NCI) plots; (**c**) Bond Critical Points (BCPs); and (**d**) Ring Critical Points (RCPs). Regarding subfigures (**a**,**b**), the isosurfaces are coloured according to the values of sign(λ_2_)ρ (a.u.), from −0.04 to 0.02 a.u. Colour online: blue represents strong attractive interactions, green indicates van der Waals interactions and red indicates repulsive/steric interactions. Regarding subfigures (**c**,**d**), the red and yellow circles show the BCPs and RCPs of the most relevant PNIPAM 3mer-water interactions obtained via QTAIM calculation, respectively.

In Figure 6a, we encounter a detailed scatter plot, meticulously displaying the probability densities of electron positioning. This graph serves as an illustrative road map guiding us through the complex choreography of electron movements, revealing the potential establishment and evolution of hydrogen bonding. The substantial evidence of these essential bonds is embroidered in the scatter of data points across the plot, reinforcing the presence of significant hydrogen bonding between PNIPAM and the water molecule. Figure 6b introduces us to the RDG of our chosen system, which is suitably denoted as p6. The RDG acts as a magnifying lens for the magnitude and directionality of interatomic interactions within our molecular ensemble. The RDG isosurfaces unveil a greenish-bluish zones zone between the water molecule’s hydrogen atoms and the PNIPAM’s architectural backbone.

Our quest for bonding clarity takes a crucial turn in Figure 6c, where we focus on the computation of BCPs via the MultiWFN code to study QTAIM analysis. These BCPs predominantly appear between the oxygen atom in the PNIPAM structure and the hydrogen atoms of the water molecule, an alignment that supports our evolving understanding of the hydrogen bonding dynamics. The values for the electron density (∇ρ) and its Laplacian (∇^2^ρ) at these BCPs, neatly compiled in Table 1, are pivotal to our analysis. The QTAIM interpretation is as follows: (i) a positive Laplacian (∇^2^ρ > 0) indicates a dominance of kinetic energy density over potential energy, typically associated with closed-shell interactions and the depletion of electron density; (ii) a negative Laplacian (∇^2^ρ < 0) reflects a dominance of potential energy density, indicating the accumulation of electron density and covalent-like bonding; and (iii) for hydrogen bonds, a typical diagnostic range for ∇^2^ρ is 0.020 < ∇^2^ρ < 0.140 a.u. [32].

In Figure 6d, we show the RCPs, underscoring another facet of bonding mechanisms. An observation emerges here: the robust RCPs are seen to occur above the junction region. The spatial orientation of these RCPs suggests that the steric hindrance might prevent the water molecule from approaching the polymer structure with a higher binding energy.

**Figure 7:**
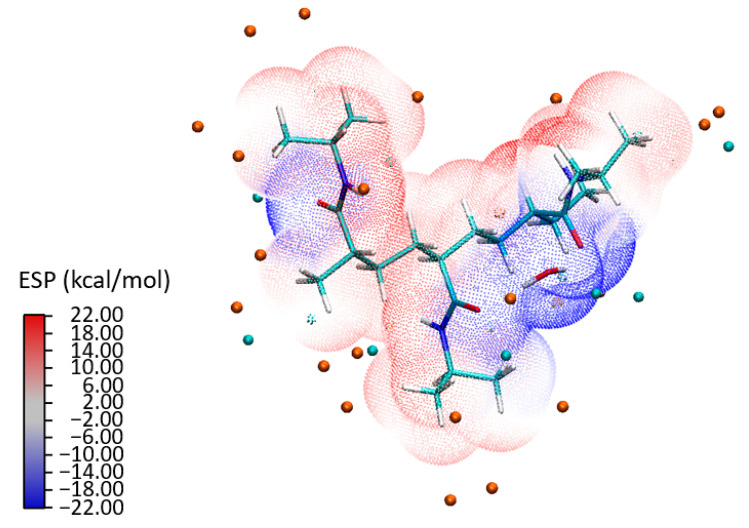
Visual depiction of ESP for the PNIPAM 3mer with a bound water molecule; the figure highlights the areas of negative potential (blue) and positive potential (red), with the water molecule predominantly located in the electron-rich (blue) region.

Figure 7 presents the ESP landscape surrounding a PNIPAM 3mer in the presence of water molecules. Here, areas of negative and positive ESP are clearly demarcated, represented by blue and red colors, respectively. It was observed that the preference of water molecules for the blue areas—regions with a negative ESP—suggests that the water molecules are likely forming hydrogen bonds or engaging in favorable electrostatic interactions with specific regions of the PNIPAM 3mer. Another important detail is the absence of areas where red and blue colors transition smoothly into each other in regions where water molecules are present. This indicates a sharp distinction between the negative and positive potential regions, possibly directing the specific orientations and interactions of water molecules with the 3mer structure. Furthermore, the blue areas near where the water molecules are located suggests that these regions are facilitating the formation of a dense shell of water molecules, likely through hydrogen bonding.

### 3.4. Analysis of Multimolecular Water Interactions with 3mer PNIPAM Structure

Elucidating upon the findings related to the 3mer PNIPAM structure and its interactions when exposed to more than a single water molecule, we broadened our focus toward studying a more complex scenario. To elucidate this, we contemplated the introduction of six water molecules at two designated locations, situated on the top and bottom ends of the PNIPAM structure. The rationale for the placement of these water molecules was informed by our prior investigations, which had highlighted the proximity of the junction zone as a potential hotspot [33,34].

**Figure 8:**
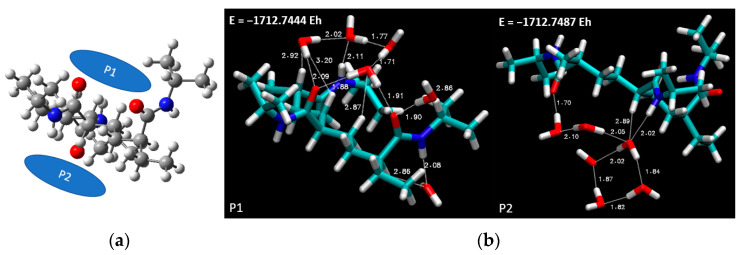
(**a**) Spatial placement and (**b**) energy optimization of six water molecules in proximity to the 3mer PNIPAM structure.

In Figure 8a, the selected spatial positions are lucidly portrayed. This figure provides a visual representation of the location of the six water molecules relative to the PNIPAM structure. This arrangement was subsequently subjected to DFT simulations using the previously selected BLYP theory level and the 6-311++G** basis set. The ensuing optimized overall energies are also depicted in Figure 8b, offering a clear perspective on the energy landscape of the system under consideration.

**Figure 9:**
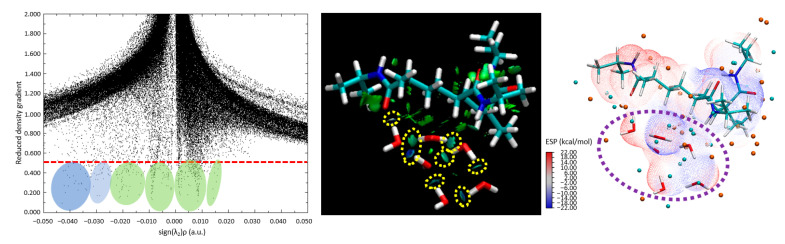
(**left**) Scatter plot of the RDGs, (**center**) visual interpretation of the RDGs, and an (**right**) ESP isosurface analysis of the PNIPAM 3mer case surrounded by the water molecules case.

Progressing with the most favorable conformation, corresponding to the case denoted as P2, which boasts the lowest overall optimized geometry energy (−1712.7487 E_h_), we further deepened our analysis. Figure 9 includes a scatter plot, RDG, and ESP maps, all of which affirm the viability of robust hydrogen bonding, even in the presence of an aggregate of water molecules. Crucially, the observations gathered hint toward the propensity of water to persist as a cluster, and not merely as isolated molecules, in proximity to the PNIPAM 3mer structure. Thus, this work not only enhances our comprehension of the interaction dynamics at play but also enriches our understanding of the intricate molecular-level phenomena underpinning these systems.

In Figure 9, it is evident from the scatter plot (left panel) that the tails are formed on the plot below the critical threshold to observe strong H-bonding formations, and they are highlighted with green and blue circles. The center panel in Figure 9 is the visual representation of the RDG. The blue and dark green isosurface regions between the water molecules and the polymer indicate that strong H-bond-like interactions are being established. The yellow dashed circles indicate the area where strong hydrogen bond-like interactions are captured as isosurfaces through RDG analysis. In this figure, we can observe that certain water molecules remain bonded to other water molecules, forming a hydrogen-bond network. However, these molecules tend to distance themselves from the polymer structure. This behavior is also supported by the ESP diagram (Figure 9, right panel), where some water molecules fall within the blue negative potential surfaces, and some fall just at the borderline or transition region of positive to negative potentials. This shows indications of molecular-level saturation limits of the studied 3mer structure.

### 3.5. Scrutinizing the Saturation Capability of the PNIPAM Backbone and Elucidating the Implications of Water Molecule Interactions

The following section engages with an in-depth scrutiny of the saturation capability of the PNIPAM backbone, specifically the segment that displays pronounced characteristics of water sorption. This segment is zoomed in on, extracted, and studied in isolation to accentuate the behavior of this critical part of the polymer.

**Figure 10:**
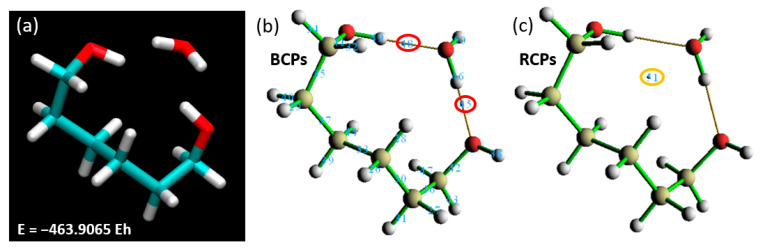
Total energy for a single water molecule interacting with (**a**) the isolated PNIPAM segment and the corresponding Laplacian of electron density at (**b**) BCPs and (**c**) RCPs.

To simplify simulation work and reduce computational time, focusing solely on a specific section of a larger compound is a recognized strategy [35,36]. An isolated segment was detached from the rest of the structure for further investigation. and its terminal atoms were hydrogenated, a widely accepted methodology in such simulation scenarios. Commencing with the application of our established theory level and basis set (BLYP with the 6-311++G** basis set), we endeavored to delineate the binding energy when this isolated segment interacts with a single water molecule. The outcome of this analysis yielded a total energy value of −463.9065 EH (Figure 10), presenting an intriguing beginning to our investigation.

**Figure 11:**
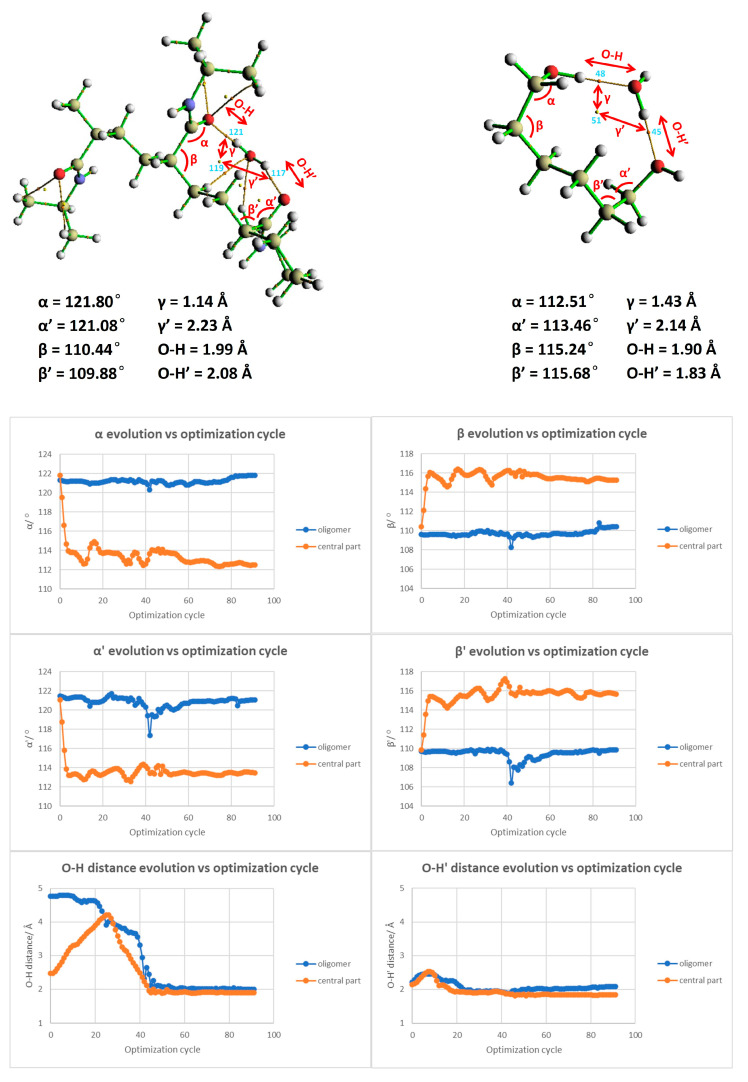
Comparative analysis of the evolution of geometric parameters in the full PNIPAM 3mer structure and the isolated linker section.

Following the calculation of the binding energy, our analysis pivoted toward discerning the ring and BCPs. The Laplacian of electron density, computed at BCP45 and BCP48, registered values of 0.10910 and 0.09615 a.u., respectively. This unveils a strong interaction between the water molecule and the hydrogenated polymer segment, potentially suggestive of hydrogen bond formation. Reinforcing this observation, RCP51 yielded a value of 0.00884 a.u., buttressing our prior inference drawn from Figure 9 (right)—the presence of a steric hindrance at the linker restricts the proximity of water molecules to PNIPAM and hampers the accretion of more water molecules over the surface. To ascertain the viability of focusing only one fragment of the polymer and validate the focused analysis of the linker backbone of the polymer, we delved into critical geometric parameters, as depicted in Figure 11. We have pinpointed essential parameters to monitor the geometric and morphological alterations throughout the simulation. These parameters, including angles, dihedrals, and the crucial distances between the water molecule and the primary interaction site on the polymer segment, are depicted in Figure 11. Additionally, we present a comparison of these critical parameters between the large-scale oligomer and the smaller-scale or extracted segment from the polymer at the top of Figure 11. Our exploration revealed an interesting finding: the evolution of angles and dihedrals does not show dramatic changes when comparisons are drawn between the full PNIPAM 3mer structure and the middle section of the linker. The lone exception was the case of the α’ angle, which registered a significant deviation between the two. This divergence is explainable due to the flexibility instilled by the hydrogenated ends.

**Figure 12:**
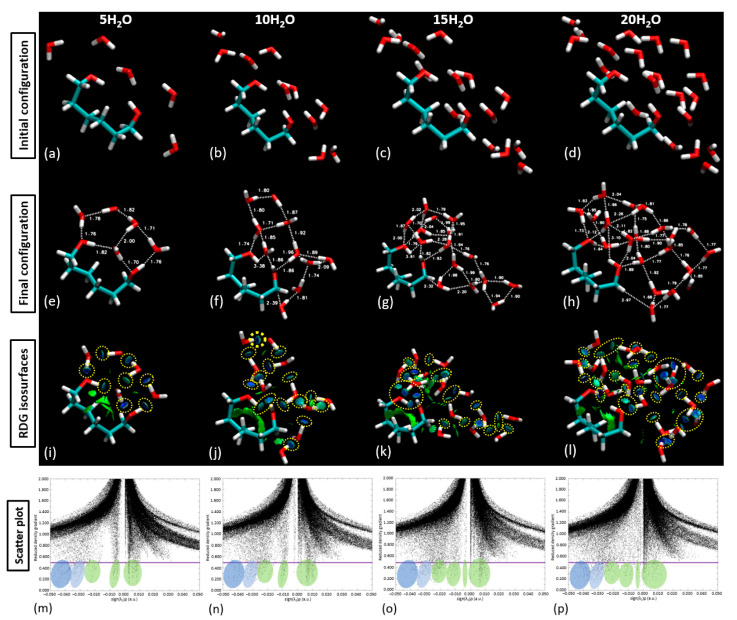
Sequential population of the central part of the PNIPAM linker with increasing quantities of water molecules (5, 10, 15 and 20 H_2_O). (**a**–**d**) Initial configurations showing the random spatial distribution of water molecules around the PNIPAM linker; (**e**–**h**) Final configurations after DFT calculations, illustrating the formation of hydrogen bonding networks between water and PNIPAM. Selected bond distances (in Å) highlight interaction strengths and structural reorganization; (**i**–**l**) RDG isosurfaces revealing noncovalent interactions (green for van der Waals, blue for hydrogen bonding, and red for repulsive forces). Yellow dotted circles indicate key interaction regions; and (**m**–**p**) Scatter plot of the RDGs corresponding to each system, showing an increase in attractive and van der Waals interactions with rising water content, indicating a pseudo-solubility threshold near 15–20 H_2_O.

With the validation of our approach, we proceeded to populate the central part of the linker with 5, 10, 15, and 20 water molecules, as demonstrated in Figure 12. This figure compares the initial and final configuration of the water saturation runs over the PNIPAM linker/central structure. Upon the examination of the RDG isosurfaces and scatter plot analysis, it was evident that in the scenarios of 5 and 10 water molecule placements, the water molecules remained as a cluster atop the polymer structure. Figure 11 presents an in-depth exploration of the geometric parameter evolution in both the entire 3mer PNIPAM structure and its isolated linker section. By using a comparative approach, this figure allows us to appreciate the individual contributions of the linker section and its synergistic influence when it is a part of the more extensive 3mer structure. To understand this figure more thoroughly, it is vital to identify the core objective: to reveal how the linker section and the entire 3mer structure respond to incremental introduction of water molecules, focusing on variations in their geometric parameters. This allows us to discern the role of hydration in influencing the geometric and, by extension, the physical properties of the PNIPAM structure.

In the case of the isolated linker section, the geometric parameters demonstrate a consistent trend up to the addition of 10 water molecules, indicative of a stable interaction system with a somewhat linear response to hydration. The relatively steady trend suggests that the linker section can comfortably accommodate up to 15 water molecules without significant alterations to its geometry. However, upon the addition of the 16th (up to the 20th) water molecule, there is a noticeable deviation in the geometric parameters. When the 15th–20th water molecules were introduced, around six water molecules began to drift away from the polymer, signifying a weakened ability of the polymer to retain them. This observation suggests a limit on the number of water molecules that can be effectively coordinated by the polymer, approximated at 15 above the linker. The result is perfectly aligned with some experimental studies found in the literature obtained through high-frequency dielectric relaxation techniques [37,38], which report 11 hydrated water molecules per monomer unit below the LCST. This limitation provides a valuable reference for molecular dynamics (MD) studies and experimental comparisons to enhance our understanding of the water-binding capacity of PNIPAM. This disruption could be attributed to a saturation point where the linker section has maximized its water retention capacity. This observation uncovers a potential threshold for water sorption for the isolated linker section, beyond which the system’s geometric stability is affected. When considering the full 3mer structure, the impact of the hydration process becomes even more nuanced. We consider that to be the water sorption capacity of the complete 3mer structure, providing an intriguing comparison point with the isolated linker section. It seems that the more complex the structure, the more water molecules it can stably accommodate, a factor that may be attributed to the increased number of interaction sites within the structure. By comparing the isolated linker section with the full 3mer structure, Figure 12 serves a dual purpose: it pinpoints the pseudo-saturation point for water sorption for each configuration and provides invaluable insights into the differential response of the structures to hydration. Furthermore, this comparison underlines the remarkable ability of the PNIPAM structure to adapt to incremental hydration, thereby spotlighting the polymer’s potential for various hydration-sensitive applications.

### 3.6. Unveiling the Interaction Dynamics Between Water Clusters and Heptameric PNIPAM: An Exploration of the Interior and Exterior

**Figure 13:**
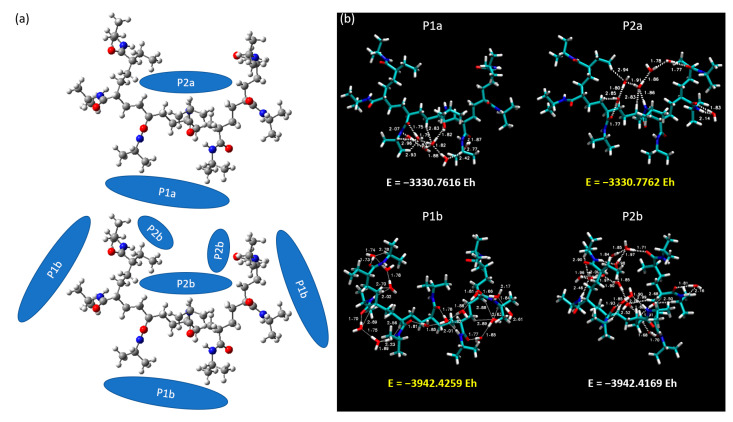
(**a**) Spatial arrangements of water molecule clusters within and around the 7mer PNIPAM structure, and (**b**) case studies P1a, P2a, P1b, and P2b.

In this section, we focus our investigative prowess on the intricate interactions between a heptameric (7mer) PNIPAM structure and clusters of water molecules. The complex structural dimensionality of a 7mer system permits the exploration of interactions in a more diverse set of circumstances, including an exploration of interactions within the interior as well as around the exterior of the PNIPAM construct. In Figure 13, we provide a detailed visual representation of the two principal arrangements of water clusters in relation to the 7mer PNIPAM system. These configurations, denoted as “a” and “b”, strategically encapsulate the two primary settings under investigation: the interior and exterior regions of the 7mer architecture. In the configurations classified as P1a and P2a, we focused on clusters of six water molecules. This selection is deeply rooted in our previous studies and lends continuity to our investigations, allowing us to extrapolate our findings to more complex systems. Conversely, in the P1b and P2b configurations, we increased the scale of our investigation to encompass 14 water molecules. This number was not selected arbitrarily but was a result of a calculated pseudo-saturation value derived from our previous comprehensive investigations.

Our investigations revealed intriguing outcomes, with scenarios P2a and P1b recording higher total energies at −3330.7762 EH and −3942.4259 EH, respectively, as depicted in Figure 13. As a keen observer might anticipate, the b configurations inherently present higher binding energies due to the increased number of water molecules interacting with the PNIPAM structure.

**Figure 14:**
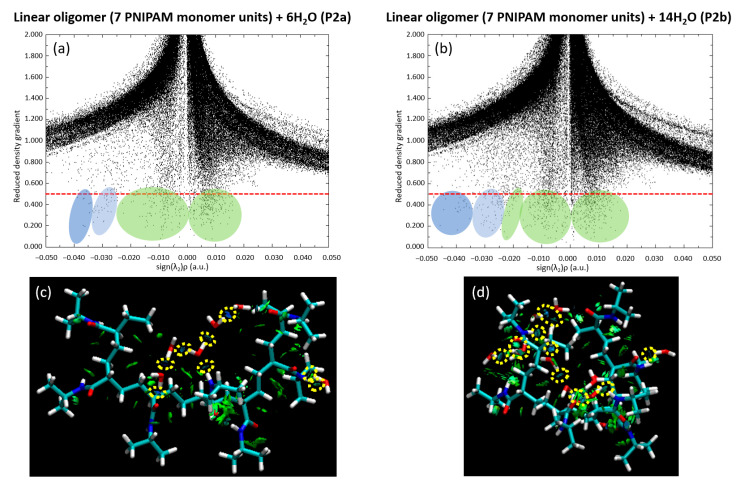
Decoding the PNIPAM–water interaction: scatter plot of configurations (**a**) P2a and (**b**) P2b, and RDG analysis (**c**) P2a and (**d**) P2b.

We further extended our investigation by analyzing the scatter plot and RDG for both the P2a and P2b configurations. These analyses, visually presented in Figure 14, deepened our insights into the dynamic relationship between the water clusters and the 7mer PNIPAM structure. We specifically chose the P2b configuration due to its highest binding energy. Additionally, we selected P1b as it complements the P2b selection, offering a perspective on water interactions from the side of the polymer. Figure 14 elucidates the intricate dynamics between water clusters and the 7mer PNIPAM structure, with a particular emphasis on configurations P2a and P2b.

Notably, a preponderance of water molecules exhibits a tight clustering pattern in the plot, implying a strong bonding environment akin to hydrogen bonding shared among them. This observation is evident in both the P2a and P2b configurations, validating the ubiquitous role of hydrogen bond-like interactions in defining the water–PNIPAM system dynamics. The RDG analysis, on the other hand, provides a holistic view of the electron density within the system. In configurations P2a and P2b, the RDG values show a marked consistency, further reinforcing the inference of robust hydrogen bonding drawn from the scatter plot. Furthermore, the RDG data corroborates the presence of steric hindrance within the P2a configuration, an intriguing observation indeed. Steric hindrance, caused by the physical presence of atoms or groups of atoms restricting the path of other groups within the molecule, could be considered a potential disruptor of interaction strength. However, the analysis shows that, while steric hindrance is indeed present, it does not lead to any significant destabilization of the system. This suggests that the 7mer PNIPAM structure, with its complex conformation, can still maintain stability amidst slight geometric tensions, a testament to its architectural resilience.

Upon closer examination of the P2b configuration, a discernible decrease occurs in interaction strength between certain water molecules and the polymer. Despite this reduction, these water molecules still maintain their overall integrity as a cohesive cluster, behaving in a way that is reminiscent of a bulk phase. This observation has pivotal implications, especially in the context of studying larger PNIPAM structures and their interactions with water clusters. It suggests the possibility of water clusters preserving their structural identity, even when in association with larger polymers, possibly paving the way for intriguing macroscopic behaviors.

**Figure 15:**
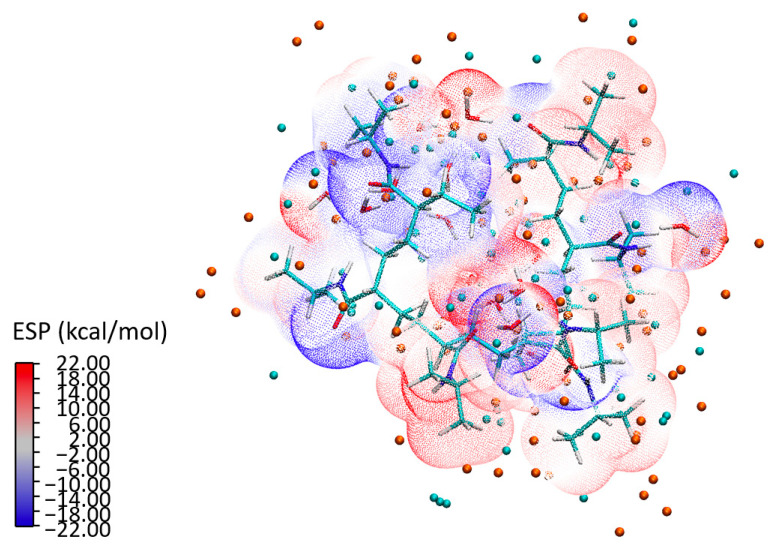
ESP isosurface for configuration P2a: an in-depth insight into electron distribution and bond formation.

To build a holistic understanding of these interactions, we conducted a detailed analysis of ESP, illustrated in Figure 15 for the P2a configuration. The isosurface representation of the ESP depicts a scattering electron distribution across the 7mer macrostructure. The distinct separation between the water clusters and the PNIPAM structure—as seen in the ESP—underlines the formation of strong bonds. The analysis not only shows a combination of individual atomic potentials, but rather showcases how these potentials influence and shape each other, leading to a unique electronic topography that is emblematic of the PNIPAM–water interaction. The isosurface representation in Figure 15 reveals a scattering electron distribution that reflects the electronic reorganization occurring within the system. The electron density is dynamically redistributed in response to interactions with neighboring atoms. Notably, the ESP shows a distinct spatial separation between the water clusters and the PNIPAM structure. It is evident that the structure of the polymer and the water clusters foster a cooperative environment that encourages the formation of robust bonds, thereby highlighting the innate compatibility of the water clusters and the PNIPAM structure. This distinct separation, as seen in the ESP, underlines the strong interaction between the entities, leading to an efficient transfer of charge and, ultimately, the formation of strong, stable bonds.

These findings are further corroborated by a meticulous exploration of BCPs and RCPs, with the data succinctly presented in Table 2. The systematic investigation of these critical points enhances our understanding of the bonding behaviors, bringing us one step closer to the comprehensive characterization of the PNIPAM–water interaction.

### 3.7. Molecular Dynamics (MD) Results and Hydration Dynamics of PNIPAM Across the LCST Threshold

#### 3.7.1. Radius of Gyration Insights

**Figure 16:**
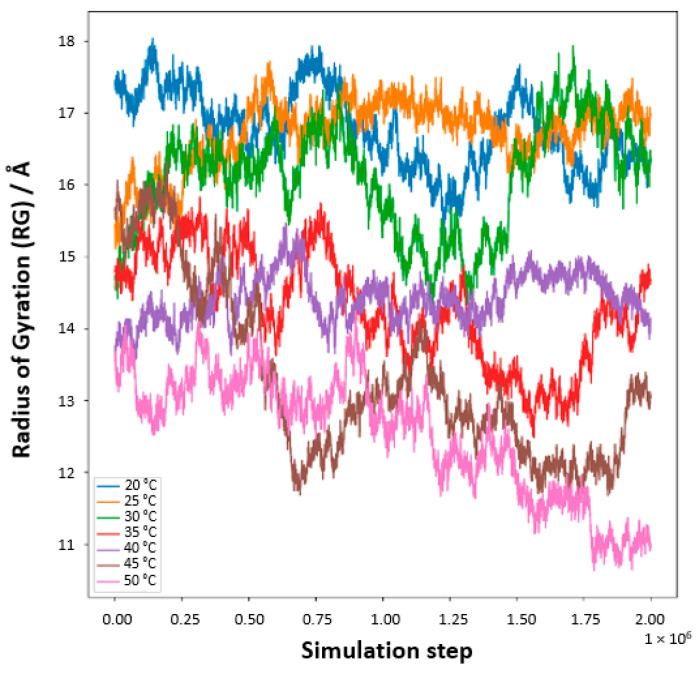
Radius of gyration, *R*_g_, of an OPLS-AA PNIPAM 30mer chain in aqueous solution (5000 SPC/E water molecules) at seven temperatures as a function of time.

Our meticulous exploration into the temperature-responsive behavior of PNIPAM in water solutions unveils a pronounced temperature-dependent modulation of the polymer’s radius of gyration (*R_g_*, Figure 16). At reduced temperatures, specifically 20 °C and 25 °C, the *R_g_* values surge, signifying an expanded state of the PNIPAM chain. This expansion ostensibly results from enhanced hydration, where the polymer adopts an extended conformation within the aqueous domain. The elevated *R_g_* values are emblematic of a well-solvated and unfurled chain, predominantly at these lower temperature conditions. As the thermal conditions are escalated, particularly approaching the 30 °C to 35 °C range, a conspicuous diminution in *R_g_* values is observed. This reduction symbolizes a contraction of the PNIPAM chain, a phenomenon that is resonant with the polymer’s LCST behavior. The chain’s transition from a hydrophilic to a hydrophobic nature is marked by a collapse, aligning with the LCST concept where PNIPAM exhibits a phase transformation. Ascending further into the thermal scale, temperatures surpassing 40 °C mark a continued decline in *R_g_* values, delineating a transition into a globular, more condensed state of the polymer. This morphological alteration is attributed to the ejection of water molecules concomitant with the temperature elevation past the LCST, heralding a compacted state of the chain.

**Figure 17:**
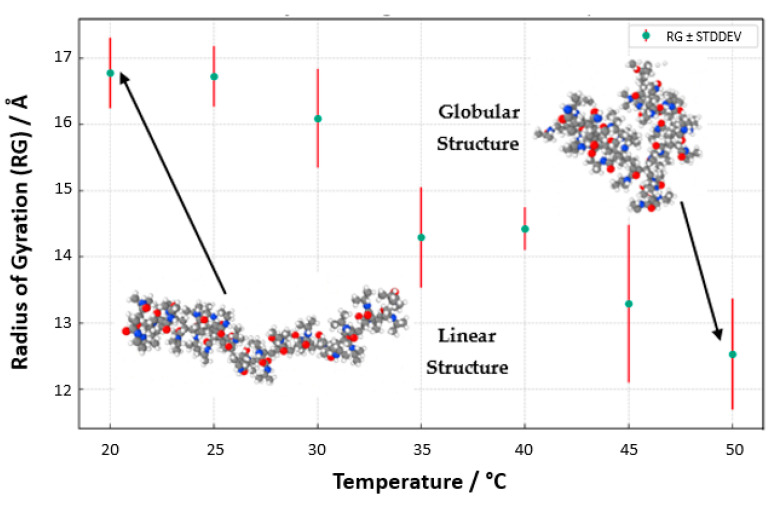
Radius of gyration, *R*_g_, of an OPLS-AA PNIPAM 30mer chain in aqueous solution (5000 SPC/E water molecules) vs. the temperature.

To better visualize the geometry of the structure, we averaged the *R_g_* values through the structure relaxation period, as shown in Figure 17 (*R*_g__averaged vs. temperature with standard deviation). The aggregation of these data points, with associated standard deviations as error bars, presents a compelling narrative of the structural evolution of PNIPAM, echoing the delicate interplay between temperature and polymer solvation. Regarding conformational properties, it is also worth mentioning that this decrease in *R_g_* values from the coil state to the globule state as temperature increases is consistent with some experimental studies found in the literature using small-angle X-ray scattering (SAXS) [39], which report an *R_g_* of 6.0 ± 0.3 nm for a linear PNIPAM of 92 kg/mol in the coil state and 2.2 nm for cyclic PNIPAM in the globule state. Furthermore, our simulations capture the coil-to-globule transition around 32 °C, corroborating experimental observations of PNIPAM’s LCST behavior.

#### 3.7.2. Radial Distribution Function Elucidations

**Figure 18:**
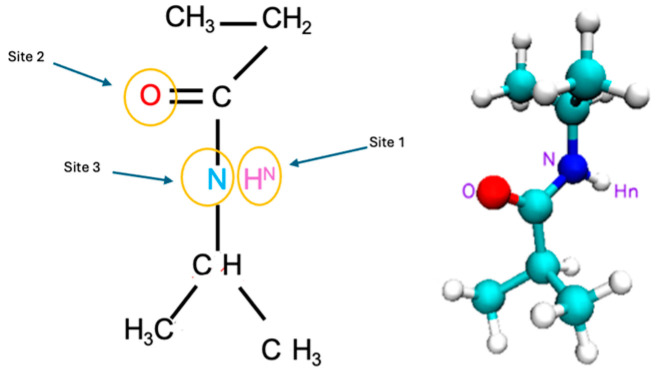
One PNIPAM unit structure shown at the RDF studied sites.

We examined the nuances of specific interaction sites within the PNIPAM and water system to point out the most effective sites that lead the water sorption. The targeted sites are shown in Figure 18 and they include *(i)* the [H^N^ PNIPAM_O water] site probing into the amide hydrogen atom of the PNIPAM and its affinity with the oxygen atom in water, *(ii)* the [O PNIPAM_H water] site investigating the carbonyl oxygen of PNIPAM and its interactions with hydrogen in the water molecule, *(iii)* the [N PNIPAM_O water] site exploring the proximal relationships between water’s oxygen and PNIPAM’s nitrogen atom, and *(iv)* the [N PNIPAM_H water] site investigating the hydrogen bonding dynamics between water’s hydrogen and PNIPAM’s nitrogen atoms. These sites were selected as each of them offers a unique window into the complex hydration phenomena and temperature-dependent behaviors of PNIPAM, elucidating the intricate interplay of molecular interactions that govern the system’s physicochemical properties.

**Figure 19:**
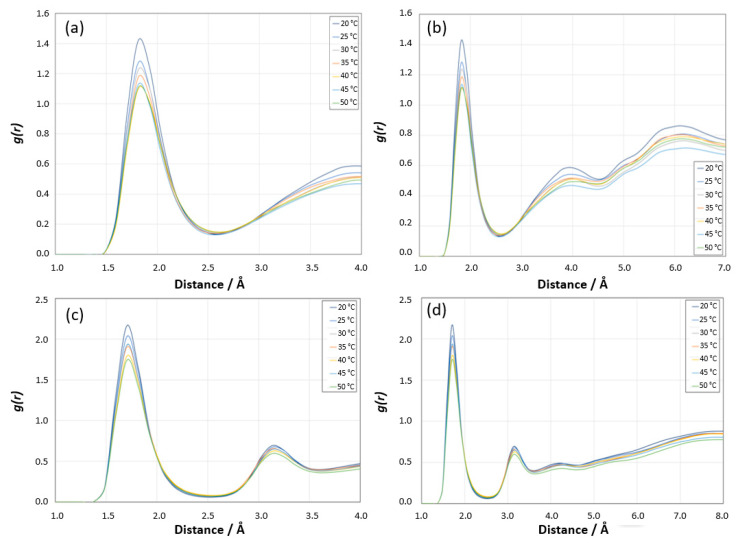
(**a**) Radial distribution function *g*(*r*) showing H^N^_O site of PNIPAM–water coordination at different temperatures and 0–5 Å distance range, (**b**) 0–7 Å distance range. (**c**) Radial distribution function *g*(*r*) showing O_H site of PNIPAM–water coordination at different temperatures and 0–5 Å distance range, (**d**) 0–8 Å distance range.

Radial Distribution Function Analysis for [H^N^ PNIPAM _O water] site: The first sharp peak around 2 Å is indicative of strong interactions between the hydrogen atoms of PNIPAM and the oxygen atoms of water, which is typical of hydrogen bonding, as shown in Figure 19a. The position of this peak is consistent with the expected distance of a hydrogen bond. The height of the first peak decreases with increasing temperature. At lower temperatures (20 °C and 25 °C), the higher peak suggests stronger or more hydrogen bonding, consistent with the more hydrated state of PNIPAM at these temperatures. As the temperature increases, the reduction in peak height indicates weaker or fewer hydrogen bonds, which correlates with the decreased hydration of PNIPAM upon approaching and surpassing its LCST. The behavior in the intermediate distance range (approximately 3–5 Å) shows less variation with temperature compared to the first peak, but still suggests some temperature-dependent changes in the local structure or solvation shell around the PNIPAM. Beyond 5 Å, the *g*(*r*) curves for different temperatures tend to converge, indicating that long-range order is less affected by temperature changes in this system, as shown in Figure 19b. This region reflects the structure beyond the immediate solvation shell, where the temperature effects on the polymer–water interaction are less pronounced. The shape and behavior of the RDF suggest that the PNIPAM’s hydration shell structure changes with temperature, which is typical for polymers exhibiting LCST behavior. At lower temperatures, PNIPAM is well solvated, and as the temperature increases, the solvation structure becomes less defined, reflecting the transition from a hydrophilic to a hydrophobic state.

Radial Distribution Function Analysis for [O PNIPAM_H water] site: A pronounced first peak appears at around 1.8 Å, and this peak represents the strong interaction between the amide oxygen of PNIPAM and hydrogen of water molecules, as shown in Figure 19c. Similarly to the previous RDF for the hydrogen of PNIPAM and the oxygen of water, the height of this peak decreases with increasing temperature, which is consistent with the more hydrophilic nature of PNIPAM below its LCST. The sharpness and slight shift in the position of the peak towards lower *r* values with increasing temperature might indicate a change in the nature of the hydrogen bonding as the system approaches and exceeds the LCST. The *g*(*r*) beyond the first peak up to about 5 Å shows some variability with temperature, indicating changes in the solvation shell structure around the PNIPAM, as shown in Figure 19d. Beyond this range, the curves begin to overlap, suggesting that at larger distances, the effect of temperature on the structure of the water around the polymer is minimal.

**Figure 20:**
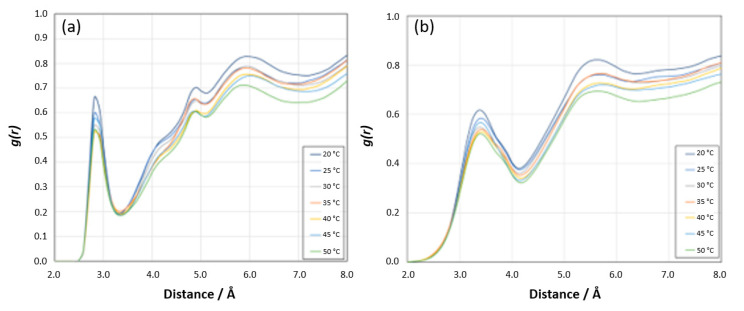
(**a**) Radial distribution function *g*(*r*) showing N PNIPAM_O water site coordination at different temperatures and 0–8 Å distance range. (**b**) Radial distribution function *g*(*r*) showing N PNIPAM_H water site coordination at different temperatures and 0–8 Å distance range.

Other Sites: The RDF analyses for the [N PNIPAM_O water] and [N PNIPAM_H water] sites demonstrate relatively lower intensity peaks compared to the [H^N^ PNIPAM _O water] and [O PNIPAM_H water] sites (Figure 20a,b), suggesting subtler interactions at these sites. At lower temperatures, the first peaks before 3 Å reveal the proximity of water molecules to the nitrogen atoms of PNIPAM, indicative of a defined hydration shell which diminishes as temperatures increase, aligning with the expected LCST behavior of PNIPAM. As the temperature rises, these interactions wane, reflecting a decrease in hydration and a transition from hydrophilic to hydrophobic states. Beyond 5 Å, the RDF profiles converge towards a pattern resembling bulk water, implying that at these distances, the structure of water is largely unaffected by the presence of PNIPAM. This subdued variation underscores the lesser role these sites play in the overall hydration dynamics of PNIPAM.

## 4. Conclusions and Future Work

In conclusion, our study has provided profound insights into the mechanisms of interaction between water molecules and PNIPAM structures of varying complexity, from a monomer to a heptamer, employing a gamut of sophisticated computational methodologies. Our study elucidates the unique interplay of hydrogen bonding and steric hindrance in defining the interactions between water molecules and the PNIPAM construct. We have deciphered the crucial role of the linker zone in the polymer and identified an approximate pseudo-saturation limit for water sorption onto the structure based on the examination of the initial and final configurations, the RDG isosurfaces and the scatter plot analysis of the central part of the PNIPAM linker with increasing quantities of water molecules. Furthermore, we have explored the intriguing spatial arrangement of water clusters both within and around the 7mer PNIPAM structure and uncovered the pivotal role of electron distribution in governing these interactions. To assess the quantitative impact of steric hindrance at the linker region on water binding kinetics, both the spatial proximity and persistence of hydrogen bonds between water molecules and the linker atoms have been examined during MD trajectories. The region exhibits limited conformational flexibility and electron density crowding, as evidenced by QTAIM and RDG analyses. This crowding introduces geometric constraints that reduce the frequency and residence time of water molecules near the sterically hindered sites, particularly beyond 15 coordinated molecules. These observations suggest that the steric bulk of the isopropyl groups and their spatial arrangement above the backbone impede further hydrogen bond formation, thereby acting as a kinetic barrier to additional water sorption. While the linker remains chemically active, steric effects impose a practical upper bound on hydration.

The MD simulation studies complement the static picture obtained from DFT by offering a dynamic view of PNIPAM’s hydration. The total 20 ns run was found to be enough for observing the equilibrium hydration behavior and structural fluctuations, as evidenced by the stabilization of key metrics such as total energy, temperature and the radius of gyration (*R_g_*) within 10–15 ns in the temperature range studied. While simulations beyond 20 ns could potentially offer additional insight into long-timescale fluctuations, the key hydration features and saturation behavior reached steady-state conditions over the last 5–10 ns of each trajectory. Through the temperature-dependent analysis of the *R_g_*, MD simulations show how the polymer chain’s conformation changes with temperature, suggesting where on the polymer these interactions are strongest. The radial distribution function (RDF) further refines this dynamic picture, demonstrating how the proximity and intensity of water–polymer interactions vary with temperature, particularly at key sites identified by DFT. Both DFT and MD highlight the amide hydrogen ([H^N^ PNIPAM_O water] site) and the carbonyl oxygen ([O PNIPAM_H water] site) sites as prominent interaction sites. DFT calculations show that these sites have high probabilities of electron density conducive to hydrogen bonding, while MD simulations observe significant alterations in hydration dynamics at these sites as temperature changes. For instance, both methodologies suggest that at lower temperatures, these sites exhibit stronger hydrogen bonding, which corroborates the well-hydrated and expanded state of PNIPAM observed in MD simulations. As the temperature increases, both DFT and MD note a weakening of these interactions, consistent with the transition to a hydrophobic state beyond the LCST. The observed conformational collapse and reduction in hydration above 32 °C is in strong qualitative agreement with the experimentally reported LCST of PNIPAM, thereby validating the temperature responsiveness captured in our MD simulations. In contrast, the amide nitrogen exhibited a weaker interaction with water, likely due to both electronic and geometric factors. From a quantum chemical perspective, the amide nitrogen is partially shielded due to resonance delocalization within the amide group, which reduces its availability as a hydrogen bond acceptor. In contrast, the carbonyl oxygen and the amide hydrogen are more accessible and exhibit stronger partial charges, making them more favorable for directional hydrogen bonding with water. Additionally, steric hindrance and spatial positioning reduce the likelihood of water accessing the lone nitrogen pair. These effects were evident in our ESP and QTAIM analyses, which showed less localized electron density and fewer bond critical points near the nitrogen compared to the oxygen and hydrogen sites. Although solvent-excluded volume was not explicitly calculated, RDFs and hydrogen bond distributions suggest that local geometry imposes spatial constraints that limit water access to the nitrogen site. Regarding the persistent decrease in Rg above 40 °C, it is interpreted as a continuation of the coil-to-globule transition that is typical of PNIPAM above its LCST. This behavior reflects enhanced intramolecular hydrophobic interactions and the further compaction of the polymer chain, which has been reported in both simulation and experimental studies. Importantly, aggregation is unlikely in our system due to the use of a single polymer chain in the MD simulation box, preventing inter-chain associations. The decrease in Rg is thus attributed to conformational collapse within the individual chain, not multi-chain aggregation. This interpretation is supported by RDF analysis and hydrogen bonding trends, which indicate reduced hydration and strengthened intra-chain contacts beyond the LCST.

Experimental studies found in the literature have shown that PNIPAM can absorb 11 water molecules per monomer unit below the LCST [37,38]. These experimental values align with the hydration numbers calculated in our work across PNIPAM structures. Such hydration levels are considered critical for assessing PNIPAM’s functionality in practical applications. In biomedical systems, the degree of polymer swelling influences drug release profiles [40,41], while in agricultural contexts, water retention and thermal responsiveness are relevant for controlling soil moisture under variable environmental conditions [42]. The temperature-dependent hydration dynamics and saturation thresholds identified herein offer predictive insights that may inform the rational design of PNIPAM-based materials for these and related applications. Moreover, the DFT-derived binding energies trends for water interacting with PNIPAM monomer and oligomer units (e.g., strong binding interactions with the amide hydrogen and the carbonyl oxygen sites, saturation behavior beyond ~15 water molecules) are qualitatively consistent with some experimental enthalpies of hydration found in the literature, thereby supporting the validity of the DFT data as a reliable proxy for interpreting site-specific hydration energetics. Dannenberg [43] reported gas-phase DFT calculations at the B3LYP/D95++(d,p) level for N-Methylacetamide (NMA) interacting with up to three water molecules. The calculated interaction enthalpy for NMA with three water molecules was approximately −14.11 kcal/mol, and after accounting for water–water interactions, the corrected enthalpy was −15.10 kcal/mol. These values reflected strong hydrogen bonding between the amide hydrogen and the carbonyl oxygen of NMA and water molecules. Tavagnacco et al. [44] also investigated the coil-to-globule transition of PNIPAM in aqueous solutions using differential scanning calorimetry (DSC). The enthalpy change (ΔH) associated with the transition was reported to be approximately 5.5 kJ/mol per repeating unit. This transition reflected the net enthalpic change due to the disruption of hydrogen bonds between water molecules and the amide groups of PNIPAM during dehydration.

The molecular-level insights gained from our study are directly relevant to the local segmental behavior of crosslinked PNIPAM gels. In gels, these hydration mechanisms operate similarly within polymer strands between crosslink junctions. However, macroscopic properties like swelling capacity, mechanical response and cooperative hydration effects are additionally governed by crosslink density and network topology, which are not captured in our present model. Therefore, the current findings provide a mechanistic foundation for understanding water–polymer interactions at the molecular scale, but further work incorporating networked structures is necessary to fully extrapolate to gel-phase behavior. Building on this molecular-level framework, our findings extend the current understanding of PNIPAM hydration and provide a foundation for future investigations and practical material design. By identifying pseudo-saturation thresholds, dominant interaction sites and temperature-sensitive hydration behavior, the findings suggest specific structural features that could be targeted to tune performance in water capture, biomedical or environmental applications. However, certain limitations remain, including the finite polymer lengths modeled, the absence of explicit polymer crosslinking, and the lack of statistical MD replicates due to computational constraints. Additionally, hydration behavior at longer time scales or under non-isothermal conditions remains unexplored. Future work should incorporate longer chains, more extensive statistical sampling and hybrid quantum-classical approaches to bridge the gap between atomistic modeling and real-world conditions. Experimental validation of the predicted hydration numbers and interaction motifs, particularly using techniques such as FTIR, NMR or calorimetry, will also be essential to support the simulation-based findings.

## Figures and Tables

**Table 1:** Electron density and Laplacian parameters for BCPs and RCPs in the trimer PNIPAM–water complex.

BCP	ρ	∇^2^ρ/a.u.	Site
117	0.01779	0.06280	O-H
121	0.02095	0.07761	O-H
123	0.00549	0.01591	H-O
46	0.00815	0.02319	H-O
**RCP**	**ρ**	**∇^2^ρ/a.u.**	
116	0.00321	0.01179	
119	0.00471	0.01687	
133	0.00256	0.01167	

**Table 2:** Detailed BCP and RCP analysis for configurations P2a and P2b.

BCP	ρ	∇^2^ρ/a.u.	Site
205	0.02416	0.08441	O-H
208	0.03132	0.10525	O-H
218	0.03086	0.10586	O-H
227	0.02349	0.07469	O-H
237	0.02620	0.09861	O-H
249	0.01493	0.03936	H-O
251	0.02425	0.08179	O-H
253	0.04094	0.13282	O-H
257	0.00871	0.02717	H-O
264	0.02425	0.08073	H-O
269	0.02997	0.10649	O-H
284	0.02655	0.09635	O-H
**RCP**	**ρ**	**∇^2^ρ/a.u.**	
215	0.00753	0.03472	
226	0.00522	0.01868	
268	0.00276	0.01024	
271	0.00462	0.01647	
278	0.00758	0.02785	
315	0.00569	0.01881	

## Data Availability

The original contributions presented in this study are included in the article. Further inquiries can be directed to the corresponding authors.
